# External Validation of Radiation-Induced Dyspnea Models on Esophageal Cancer Radiotherapy Patients

**DOI:** 10.3389/fonc.2019.01411

**Published:** 2019-12-16

**Authors:** Zhenwei Shi, Kieran G. Foley, Juan Pablo de Mey, Emiliano Spezi, Philip Whybra, Tom Crosby, Johan van Soest, Andre Dekker, Leonard Wee

**Affiliations:** ^1^Department of Radiation Oncology (MAASTRO), GROW School for Oncology and Developmental Biology, Maastricht University Medical Centre+, Maastricht, Netherlands; ^2^Velindre Cancer Centre, Cardiff, United Kingdom; ^3^Faculty of Health Medicine and Life Sciences (FHML), Maastricht University, Maastricht, Netherlands; ^4^School of Engineering, Cardiff University, Cardiff, United Kingdom

**Keywords:** radiation-induced dyspnea, esophageal cancer, chemo-radiotherapy, prognostic model, distributed learning

## Abstract

**Purpose:** Radiation-induced lung disease (RILD), defined as dyspnea in this study, is a risk for patients receiving high-dose thoracic irradiation. This study is a TRIPOD (Transparent Reporting of A Multivariable Prediction Model for Individual Prognosis or Diagnosis) Type 4 validation of previously-published dyspnea models via secondary analysis of esophageal cancer SCOPE1 trial data. We quantify the predictive performance of these two models for predicting the maximal dyspnea grade ≥ 2 within 6 months after the end of high-dose chemo-radiotherapy for primary esophageal cancer.

**Materials and methods:** We tested the performance of two previously published dyspnea risk models using baseline, treatment and follow-up data on 258 esophageal cancer patients in the UK enrolled into the SCOPE1 multi-center trial. The tested models were developed from lung cancer patients treated at MAASTRO Clinic (The Netherlands) from the period 2002 to 2011. The adverse event of interest was dyspnea ≥ Grade 2 (CTCAE v3) within 6 months after the end of radiotherapy. As some variables were missing randomly and cannot be imputed, 212 patients in the SCOPE1 were used for validation of model 1 and 255 patients were used for validation of model 2. The model parameter Forced Expiratory Volume in 1 s (FEV_1_), as a predictor to both validated models, was imputed using the WHO performance status. External validation was performed using an automated, decentralized approach, without exchange of individual patient data.

**Results:** Out of 258 patients with esophageal cancer in SCOPE1 trial data, 38 patients (14.7%) developed radiation-induced dyspnea (≥ Grade 2) within 6 months after chemo-radiotherapy. The discrimination performance of the models in esophageal cancer patients treated with high-dose external beam radiotherapy was moderate, area under curve (AUC) of 0.68 (95% CI 0.55–0.76) and 0.70 (95% CI 0.58–0.77), respectively. The curves and AUCs derived by distributed learning were identical to the results from validation on a local host.

**Conclusion:** We have externally validated previously published dyspnea models using an esophageal cancer dataset. FEV_1_ that is not routinely measured for esophageal cancer was imputed using WHO performance status. Prediction performance was not statistically different from previous training and validation sets. Risk estimates were dominated by WHO score in Model 1 and baseline dyspnea in Model 2. The distributed learning approach gave the same answer as local processing, and could be performed without accessing a validation site's individual patients-level data.

## Introduction

In radiation therapy, radical radiation doses are expected to provide better local control than lower palliative doses, however the risk of radiation-induced adverse events is increased. Clinical symptoms of radiation-induced lung disease (RILD) include dyspnea, cough, and fever, which can have a serious effect on the patient's quality of life. Approximately 10–20% of patients with lung cancer who receive (chemo)-radiotherapy developing moderate to severe symptomatic RILD (1).

Radiation-induced dyspnea (RILD in this study) is a side-effect for patients treated with high-dose thoracic irradiation. Studies have reported the predictors for radiation-induced dyspnea for lung cancer patients treated with (chemo)radiotherapy (2, 3). The risk factors for RILD include dosimetric factors, clinical factors, pathological factors and blood biomarkers (2–16). In our knowledge, there is no published study reporting the risk factors of radiation-induced dyspnea for patients with primary esophageal cancer, which might be explained by the fact that dyspnea is not routinely assessed during follow-up of esophageal cancer treatment.

The current study conducted a TRIPOD (Transparent Reporting of A Multivariable Prediction Model for Individual Prognosis or Diagnosis) Type 4 validation (17) of previously-published dyspnea models M1 (2) and M2 (3) via secondary analysis of the SCOPE1 (18, 19) dataset. SCOPE1 was a randomized controlled trial investigating the effects of chemo-radiotherapy with and without additional cetuximab in patients with esophageal cancer, including follow-up assessments of dyspnea. We quantify the predictive performance of these two models for predicting the maximal dyspnea grade ≥ 2 within 6 months after the end of high-dose chemo-radiotherapy for primary esophageal cancer. The goal of this study is to verify two hypotheses: (I) that a common thoracic RILD model may be feasible for a different index tumor and (II) that it is feasible to perform an external validation of a toxicity model between two sites via a distributed learning approach without any exchange of patient-specific records.

## Methods and Materials

### Model Development Cohorts

Patient characteristics in the development and validation cohorts are detailed in Table 1. The first radiation-induced dyspnea model (M1) (2) was developed from 438 patients with either non-small cell lung cancer (NSCLC) Stage I-IIIB or limited disease small cell lung cancer, treated with curatively-intended (chemo)radiotherapy between January 2002 till January 2007. Patients in this cohort were predominantly male (328/438, 74.8%) with confirmed NSCLC histology (292/438, 66.7%) and a spread of chemotherapy regimens (concurrent 70/438, 16%; sequential 203/438, 46%; no chemotherapy 159/438, 36%, unspecified 6/438, 1%). RILD, including dyspnea, was scored according to CTCAE (v3.0) (20) during radiotherapy (RT) and up to a maximum of 6 months after RT. A range of radiotherapy prescribed doses from 46.9 to 79.2 Gy were used, with fraction doses not exceeding 2 Gy.

**Table 1:** Patient characteristics.

**Variable**	**D1** **Maastro clinic (*N* = 438)**	**D2** **Maastro clinic** **(*N* = 259)**	**V1** **SCOPE1 (*N* = 212)**	**V2** **SCOPE1** **(*N* = 255)**
**GENDER**				
Male	328 (74.9%)	163 (62.9%)	120 (56.6%)	145 (56.2%)
Female	110 (25.1%)	96 (37.1%)	92 (43.4%)	113 (43.8%)
**AGE (YEARS)**				
	Mean 68 (SD 9)	Mean 67.5 (SD 10.1)	Mean 72.8 (SD 8.95)	Mean 72.9 (SD 9.02)
**SMOKING STATUS**				
Current smoker	77 (29.7%)	NA	NA	NA
**WHO-PS**				
0	119 (27.9%)	63 (24.3%)	110 (51.9%)	130 (50.9%)
1	223 (52.3%)	153 (59.1%)	102 (48.1%)	125 (49.1%)
≥2	84 (19.7%)	43 (16.6%)	0	0
**CCI**				
0	132 (30.9%)	No: 184 (71.0%)	NA	NA
1	128 (30.0%)	Yes: 75 (29%)		
2	95 (22.2%)			
≥3	72 (16.8%)			
Missing	0			
**CARDIAC COMORBIDITY**				
No	132(30.9%)	No: 184 (71.0%)	208 (98.1%)	252 (98.8%)
Yes	295 (69.0%)	Yes: 75 (29.0%)	2 (1.0%)	3 (1.2%)
Missing	1 (0.1%)		2 (1.0%)	None
**BASELINE DYSPNEA SCORE**				
0	NA	78 (30.1%)	197 (92.9%)	238 (93.3%)
1	NA	140 (54.1%)	10 (4.7%)	14 (5.5%)
≥2	NA	38 (14.7%)	3 (1.4%)	3 (1.2%)
Missing	NA	3 (1.1%)	2 (1.0%)	None
**DYSPNEA SCORE AFTER RT**				
0	NA	NA	135 (63.7%)	164 (64.3%)
1	NA	NA	46 (21.7%)	53 (20.8%)
≥2	NA	NA	31 (14.3%)	38 (14.9%)
Missing	NA	NA		
**FEV**_**1**_ **(%)**				
	Mean 70.0 (SD 23)	Mean 76.0 (SD 21.86)	NA	NA
**CHEMOTHERAPY**				
No	159 (36.8%)	44 (17.0%)	0	0
Yes	273 (63.2%)	197 (76.1%)	212 (100%)	255 (100%)
Missing	0	18 (6.9%)	0	0
**TUMOR LOCATION**				
Lower/middle lobe	245 (56.3%)	76 (29.3%)	NA	NA
Upper lobe	190 (43.7%)	83 (32.1%)	NA	NA
**MEAN LUNG DOSE (GRAY)**				
	13.5 (SD 4.5)	15.7 (SD 4.44)	9.8 (SD 2.8)	9.83 (SD 2.8)
Min			0.01	0.01
Max			17.9	17.9
Median			10.0	9.9
Missing			None	45 (9.80%)
**V**_**20**_ **(%)**				
	Mean 21.0 (SD 7.3)	Mean 25.5 (SD 9.9)	NA	NA

*WHO-PS, World Health Organization performance scale; CCI, Charlson comorbidity index; FEV_1_, forced expiratory volume (1s); V_20_, volume of the lung receiving ≥ 20 Gy, SD, standard deviation. D1 and D2 are development cohorts for the validated model 1(2) and model 2 (2). V1 and V2 are validation cohorts*.

A second radiation-induced dyspnea model was developed from 259 lung cancer patients treated with curatively intended chemo(radiotherapy) between 2008 and 2011, Stage I-IIIB and fractional dose ≤ 3 Gy were used to develop a second radiation-induced dyspnea model (M2) (3). These patients were treated in two hospitals, underwent PET/CT for radiotherapy treatment planning and had lung volumes delineated in the planning system. This cohort was drawn from an earlier iso-toxicity dose escalation radiotherapy trial (clinicaltrials.gov identifier NCT00572325 and NCT00573040) with maximum tumor dose not exceeding 69 Gy. This cohort was predominantly male (163/259, 62.9%) with confirmed NSCLC histology (198/259, 75.6%), received concurrent chemotherapy (148/259, 57.1%) and had no surgery prior to radiotherapy (236/259, 91.1%). Carboplatin and gemcitabine were given for sequential chemotherapy, and cisplatin and etoposide for concurrent chemotherapy. RILD, including dyspnea, was scored according to CTCAE (v3.0), by either thoracic physicians or radiation oncologists, at baseline and every 3 months following RT.

### External Validation Cohort

Two hundred and 58 esophageal cancer patients were enrolled in the SCOPE1 (18, 19) trial from 36 UK centers between February 7, 2008 and February 22, 2012. The inclusion criteria were: non-metastatic, histologically confirmed carcinoma of the esophagus (adenocarcinoma, squamous-cell, or undifferentiated carcinoma) or gastro-esophageal junction (Siewert type 1 or 2 with <2 cm extension into the stomach); selected for definitive chemo-radiotherapy by a designated multidisciplinary team; aged 18 years or older; WHO performance status 0 or 1; stage I-III disease (TNM stage 6); and esophageal tumor length < 10 cm as measured by endoscopic ultrasound. The study protocol has been published (19) and the trial was coordinated by the Wales Cancer Trials Unit (WCTU). Recruitment in SCOPE1 was halted due to futility, but follow-up of at least 24 weeks on all recruited patients was available for secondary analysis.

All patients received four cycles of cisplatin and capecitabine; two cycles were given prior to commencement of RT, and two cycles were given concurrently with RT. This chemotherapy regimen was the most commonly used for esophageal cancer treatment in the UK. Chemotherapy dose was modulated for potential hematological toxicity (based on neutrophil and platelet counts) and kidney function (based on glomerular filtrate rate). Chemotherapy cycles were also withheld for serious non-hematological adverse events until resolution to grade 0 or 1. Half of these patients were randomized to additional cetuximab for their chemotherapy.

All 3D conformal RT plans were based on contrast CT 3 mm slices, for a prescribed dose of 50 Gy in 25 once-daily fractions. The esophageal clinical target volume (CTV) was manually delineated as a 2 cm distal and 2 cm proximal expansion along the esophagus from the gross primary tumor, and a 1 cm radial expansion. The planning target volume was an additional 1 cm proximal-distal expansion from the CTV and an extra 0.5 cm radially. Lung volume receiving 20 Gy or higher was constrained to be <25% of the total lung volume.

None of the SCOPE1 patients in the validation cohort received post-RT surgery. The majority of patients were male (145/258, 56%) with either mid- or lower-esophageal tumors (226/258, 87.6%) and mean endoscopy-defined tumor length of 5.6 cm. Toxicity scoring according to CTCAE (v3.0) was carried out at baseline, during each chemotherapy cycle, at 24 weeks and then every 3 months thereafter.

### Previously Published Dyspnea Model Parameters

The model M1 (2) consisted of the following predictors: age, WHO performance status (WHO-PS) before start of RT, nicotine use (non-/ex-smoker vs. current smoker), FEV_1_ at baseline and mean lung dose in Gy. The predictors used in model M2 (3) were dyspnea score before start of RT, cardiac comorbidity, FEV_1_ at baseline, tumor location (upper vs. middle/lower lobes of lung) and sequential chemotherapy. Multivariate logistic regression analysis was performed to build M1 and M2. The coefficients used in the models are summarized in Table 2. Both models defined adverse outcomes as dyspnea grade 2 or higher within 6 months of the end of (chemo)-radiotherapy.

**Table 2:** Coefficients obtained from the multivariate logistic regression in the first (M1) (2) and second (M2) (3) dyspnea models.

**Variable**	**Model coefficients (M1)**	**Model coefficients (M2)**
Intercept	−2.2767	−1.512
**PERFORMANCE STATUS**		
WHO-PS = 1	0.28	–
WHO-PS ≥ 2	0.57	–
Current smoker	−0.45	–
Age	0.02	–
Mean lung dose	0.05	–
Baseline dyspnea	–	0.990
Cardiac comorbidity	–	0.826
Sequential chemotherapy	–	0.610
Tumor in middle/lower lung lobe	–	−0.290
Baseline FEV_1_	−0.02	−0.007

### Model Assumptions and Missing-Values Imputation

The previous M1 and M2 had been developed on, and validated in, primary lung cancer patients. However, Forced Expiratory Volume (i.e., FEV_1_), smoking status and lung tumor location (lobe) were uniformly absent from the esophageal SCOPE1 dataset. We assumed (based on the trial protocol) that all SCOPE1 patients received chemotherapy and we simulated different population scenarios for smoking status. For the model M2, we further assumed that unintended radiation dose for esophageal cancers were most analogous to RT for lung tumors in lower and/or middle lung lobes.

Since FEV_1_ was a predictor in both M1 and M2, we imputed the missing FEV_1_ measurements of the SCOPE1 patients from available data in the model M1 development cohort while blinded to the dyspnea outcome. The imputation was based on categorical regression for WHO-PS = 0, WHO-PS = 1 and WHO-PS ≥2. A statistically significant fit for FEV1 (in % of total expired volume) was found using the model:

$$\begin{array}{l} FEV1\;\left(in\;\%\right)=82.0\;if\;WHO-PS=0\\ FEV1\;\left(in\;\%\right)=74.7\;if\;WHO-PS=1\\ FEV1\;\left(in\;\%\right)=67.3\;if\;WHO-PS\;\geq 2 \end{array}$$

### Distributed Learning

**Figure 1:**
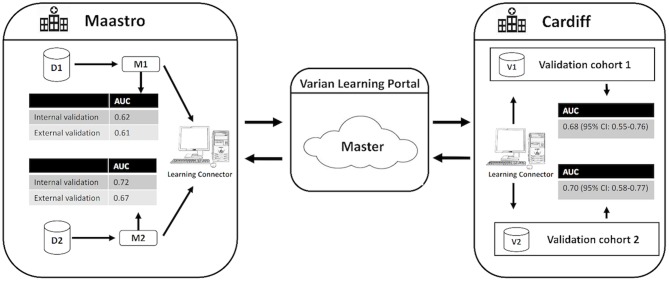
Generalized workflow of the distributed learning approach used in this study. D1 and D2 indicate the development cohorts used to develop the original RILD models M1 and M2. V1 and V2 indicate the validation cohorts for M1 and M2, respectively. CI indicates confidence interval.

External validation was performed using the same distributed methodology as published by Deist et al. (21), Jochems et al. (22) and Shi et al. (23) using the Varian Learning Portal (VLP, Varian Medical Systems, Palo Alto, CA) v1.0. A validation algorithm containing model coefficients of M1 and M2 were remotely distributed from the investigator site to the validation site via a secured http channel. The SCOPE1 data was parsed using a radiation oncology-specific semantic ontology into the Web 3.0-standard resource descriptor format (RDF). The distributed validation algorithm executes as a purely site-specific local computation by querying the local RDF repository. Only the summary classification results of validation on the SCOPE1 cohort was returned to the investigator site. Security and privacy settings within VLP blocked transfer and exposure of patient-level records from the validation site to the investigator. Previous studies (21–23) have proven that the algorithm converges to the same result as if all of the patient data was locally processed on site by an investigator. The workflow of the distributed learning approach is shown in Figure 1.

### Statistical Analysis

The validation algorithm was deployed in MATLAB, version 9.0 (MathWorks, Natick, MA). Discrimination of predictive model was evaluated using the area under the receiver-operator curve (AUC) metric (24). The AUC metric was estimated by bootstrapping (1,000 resamples). Calibration of the predictive model was assessed using calibration plots. The logistic recalibration was performed through fitting a logistic regression model by the linear predictor as the only covariate, which leads to an updated model without changing discrimination performance (25, 26).

## Results

Out of 258 available validation cases in the SCOPE1 dataset, 46 and 3 patients, respectively, were excluded from the validation due to missing values of mean lung dose for validation of model M1 and baseline scores of cardiac comorbidity and dyspnea for validation of model M2. A total of 212 patients and 255 patients were available to externally validate model M1 and M2. In the validation cohort for M1 (V1), there were 31 patients (14.3%) manifesting dyspnea grade 2 or higher within 6 months of RT. In the validation cohort for M2 (V2), 38 patients (14.9%) manifested dyspnea at the equivalent time point.

To investigate the effect of smoking status on the performance of M1 in the external validation cohort, smoking status was assigned to (i) all smokers, (ii) non-smokers, and (iii) randomly and repeat 1,000 iterations. The test yielded the AUC of 0.68 ± 0.053, 0.68 ± 0.054, and 0.65 ± 0.04, respectively by bootstrap sampling. Although the smoking status a missing predictor for esophageal validation cohort, there was no statistically significant difference in performance observed based on a bootstrapped Wilcoxon test between the three scenarios (*p* = 0.34, *p* = 0.17, *p* = 0.11). Therefore, we set it randomly in the validation cohort.

**Figure 2:**
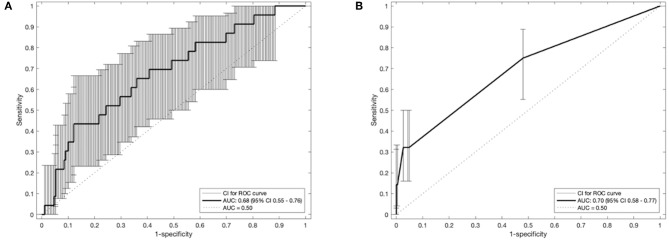
Receiver operating characteristic curves of the prognostic models **(A)**: M1 and **(B)**: M2 with 95% CI of area under the receiver-operator curve (AUC). CI, confidence interval.

**Figure 3:**
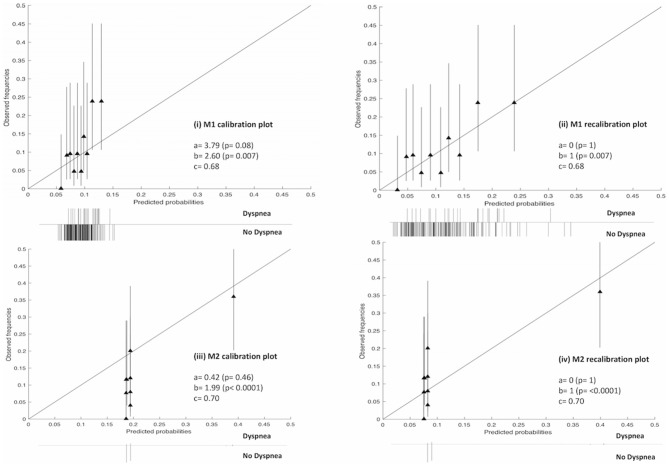
Calibration and recalibration plots of M1 and M2 on the V1 and V2 cohorts, respectively. Perfect calibration is represented by the solid line through the origin with slope = 1. Ten quantile groups were used to compare the predicted probability and the corresponding observed frequencies with a triangle. Histogram of outcomes (i.e., dyspnea or no dyspnea) is shown below each plot. a, calibration-in-the large; b, calibration slop; c, area under the receiver-operator curve (AUC).

The receiver operator curves (ROCs) of the models on external validation sets V1 and V2 are shown in Figure 2. The AUC of both models measured in the previous studies were 0.62 and 0.72 in internal validation and 0.61 and 0.67 in external validation. Compared to the previous studies, the AUC of the two models on V1 and V2 were 0.68 (95% CI: 0.55–0.76) and 0.70 (95% CI: 0.58–0.77), respectively. No statistically significant difference in performance was observed between M1 and M2 in the previous training cohorts and current external validation cohorts (AUC of M1 0.62 vs. 0.68, *p* = 0.17; AUC of M2 0.72 vs. 0.70, *p* = 0.45, Wilcoxon test). The detailed assessment of accuracy, sensitivity, specificity, positive predictive value and negative predictive value are shown in the Supplementary Table 1. Both prognostic models (M1 and M2) showed poor calibration performance and tended toward underestimation of dypsnea in the test population, which is shown in the calibration plots (Figures 3i,iii). Recalibration was performed to update the prognostic models (Figures 3ii,iv). As expected, the recalibration resulted in higher predicted risks without changing the AUCs. The calibration line of the recalibrated M1 was shifted be closer to the ideal line, whereas the calibration line of M2 was not improved overall by the recalibration.

## Discussion

The current study has tested two previously-published RILD models M1 and M2 (2, 3) on the independent validation sets V1 and V2 of the SCOPE1 trial data (18, 19), which comprises esophageal cancer patients treated with chemo-radiotherapy. Moreover, external validation was successfully implemented using an automated and decentralized approach without exchange of individual patient data.

As is well known, high-dose of thoracic radiation can often provide better local tumor control and survival for patient with cancer. Previous studies have shown that additional radiation in an appropriate range can improve locoregional tumor control and increase survival of patients with lung cancer (27–29). However, the irradiation dose in the radiotherapy treatment of esophageal cancer can have an adverse effect on lung tissue resulting in RILD, such that it leads to disutility of care and have a serious negative impact on patients' quality of life. RILD usually manifests itself in the acute (<6 months) phase as radiation pneumonitis (RP) and in the later (>6 months) phase as chronic pulmonary fibrosis (30, 31). RP is the most common dose-limiting complication of thoracic radiation with clinical symptoms such as dyspnea, cough, and sometimes fever (32). Therefore, it is a trade-off between better tumor control (i.e., better survival or lower death rate) and RILD.

The prognostic models are regarded as the basis of clinical decision support systems (CDSS) (33) that can relieve clinicians from the pressure of analyzing the large volume of publications and data by applying discoveries from research into a data-analytics architecture (34, 35). However, it is difficult to apply the results of research in clinical practice to predict which patients with esophageal cancer will likely suffer from RILD. The first reason is that many studies have investigated the risk predictors of RILD including dosimetric, clinical, pathological factors or blood biomarkers (2–16), but results between studies are highly variable or even contradictory (1, 32). In the meantime, there is no standardized lung toxicity grading system and no standard data models (so-called umbrella protocols) to guide prospective collection on routine cases. On the other hand, few publications report the risk predictors of RILD (e.g., severe dyspnea), for patients with esophageal cancer. This difficulty might be explained by the fact that dyspnea is not routinely assessed during diagnosis and prognosis of esophageal cancer.

At present, it is widely acknowledged that a prognostic model cannot be applied in clinical practice before its feasibility and practicability have been certified via validation on different levels (17, 36). External validation of a prognostic model should be performed on an/some independent cohort(s), because most models present optimistic results in the development cohorts. Validation of prognostic models involves two aspects (37). First, generalizability of a prognostic model can be described by validation on similar (reproducibility) or different (transferability) cohorts. The similarity or difference between cohorts refer to temporal, geography, methodology or investigator, which aims to distinguish from the development cohort of the original model (17, 38, 39). One primary goal of the current study to investigate the transferability of two previously-published lung toxicity models M1 and M2 under these “different” situations.

Second, accuracy performance of a prognostic model shows the statistical validity (40). Discrimination and calibration, in general, measure the accuracy performance. (i) Discrimination describes whether an individual with higher predictive probability is indeed experience RILD more often. Area under the receiver-operator curve (AUC) (24) was used to assess the discrimination performance, which is shown in Figure 2. The model M1 achieved a better discrimination performance (i.e., AUC) on V1 compared to the internal and external validation performed in the original study. The M2 obtained a better AUC on V2 than the AUC of the external validation but was consistently degraded in AUC from the internal validation of the original study. (ii) Calibration reflects the agreement between observed event and predicted risk. The calibration performance was assessed by calibration plots, which are shown in Figure 3. A perfectly calibrated model means that the predicted probabilities of RILD are identical to the observed frequencies of RILD for all patient groups. The calibration-in-the-large (i.e., intercept) of M1 and M2 were 3.79 (*p* = 0.08) and 0.42 (*p* = 0.46), and calibration slope were 2.60 (*p* = 0.007) and 1.99 (*p* < 0.0001), which indicates that predicted risks of M1 and M2 in SCOPE1 were systematically under-estimated and there was insufficient variation of covariates in V1 and V2 sets. A possible explanation may involve systematic under-reporting of clinical toxicity in the retrospectively-collected training sets. By testing different assumptions about smoking status in the test cohorts, there is no evidence to support an effect of smoking in either aggravating or protecting against dyspnea. It is also possible that the original models in lung cancer were improperly calibrated, but there was no additional information in the published articles to confirm this. However, a systematic underestimation of the dyspnea rate would be consistent with an offset error in the linear fit of FEV1 using the WHO performance score. This potential source of error could only be circumvented by measuring the FEV1 for the SCOPE1 test cases, which was not done. To correct poor calibration performance, recalibration can be performed through fitting a logistic regression model by the linear predictor as the only covariate, which leads to an updated model without changing discrimination performance (25, 26, 41). The calibration performance of M1 was moderate after conducting recalibration. The M2 model still had poor calibration performance even after recalibration, which means care should be taken applied in real clinical practice.

### Strengths of the Analysis

The SCOPE1 trial data, as an independent validation cohort, satisfied the conditions of separation in terms of temporal (different treatment time of patients in SCOPE1 and previous training cohorts), geographic (different regions, Cardiff vs. Netherlands) and investigator (different people from different institutes) from the development cohort of lung cancer. It means that the SCOPE1 was a sufficiently challenging dataset to externally validate the transferability of a prediction model between different index cancers (38, 40). Second, we have shown the RILD models (e.g., M1) can be robustly transferred to other diseased sites (e.g., esophagus) that only having the incidentally irradiated normal tissues in common without losing accuracy performance. Thirdly, this study was implemented using an automated and distributed approach without exchanging any patient data. Due to the confidentiality of patient data, local laws and technical issues, it can be prohibitively difficult to exchange patient data among hospitals. Compared to the centralized learning approach, the distributed learning approach can avoid privacy-related issues by sending research questions among institutes. The distributed learning can be achieved by transferring a machine learning algorithm to a target site and returning the results back to the sender rather than transferring real data. This process means knowledge exchange occurs without important clinical data leaving hospitals and there is no loss of validation integrity when performed distributed learning.

### Weakness of the Analysis

The current study has some limitations worthy of mention. First, some outcome data and predictor variables were missing in the validation cohorts, and data was not missing completely at random. If the missing data were compulsory predictors for the prognostic models (M1 and M2) and cannot be imputed, the corresponding patients had to be removed from the validation cohort. In addition to this, there are non-random missing data, which might be explained by the fact that the information about lung cancer were not be registered for patients with esophageal cancer in the SCOPE1 trial, such as tumor location, smoking status, and FEV_1_. For tumor location, we assumed that all of these esophageal cancer patients treated with radiation were similar to lung patients with a tumor in the lower lung lobe. For the missing FEV_1_, WHO-PS was used to impute as mentioned above. Second, there are some differences between the development (D1 and D2) and validation cohorts (V1 and V2), of which the effect on the model performance are the subject of future work. (i) SCOPE1 randomized half of the patients between cetuximab or not, whereas patients in D1 and D2 were not treated with cetuximab. (ii) All patients received chemo-radiotherapy in V1 and V2, while only 273 (63.2%) and 197 (76.1%) patients received chemotherapy in D1 and D2. (iii) The numbers of patients in D2 with baseline score 0, 1, ≥2 are 78 (30.1%), 140 (54.1%), and 48 (14.7%), whereas these numbers in V2 are 238 (93.33%), 14 (5.49%), and 3 (1.18%). It indicates that more patients had low-grade or no dyspnea overall in V2 compared with patients in D2. The effects of these uncertainties on the performance of prognostic models M1 and M2 remain unclear and are the subject of future studies.

Finally, another potential limitation is about the validated models' selection, that is the performance of M1 is moderate in terms of AUC and M2 does not include lung dose volume parameters. Although the discrimination performance of M1 is moderate, we found it achieved a similar and even better discrimination performance in the external validation cohort, which demonstrated that M1 has a good generalization. M2 was developed using multivariable regression approach. The original study (3) did evaluate mean lung dose and V20Gy as potential risk factors, but then dropped it from the final regression model because their contributions were small and/or could not be shown to be statistically significant.

### Future Work

Future work would involve two aspects. First, the M1 could be tested on a similar dataset to validate the reproducibility. Second, we would like to re-train the lung toxicity model on D1 and D2 via combining different types of features, such as image, pathological or generic features.

## Conclusion

In this study, we have externally validated previously published dyspnea models using an esophageal cancer dataset. First, the discrimination performance of the models in esophageal cancer patients treated with high-dose external beam radiotherapy are moderate, AUC of 0.68 (95% CI 0.55–0.76.) and 0.70 (95% CI 0.58–0.77), respectively. Second, risk estimates were strongly determined by WHO score in Model 1 and baseline dyspnea in Model 2. Third, the distributed learning approach gave the same answer as local validation but is feasible without accessing a validation site's patient-level data. Finally, the clinical contribution of the dyspnea prognostic model is that it would help doctors to identify patients who will likely suffer from severe dyspnea and who could therefore benefit from dose de-escalation in (chemo)-radiotherapy. Although we cannot conclude that a common thoracic RILD model is feasible for a different primary tumor, it can be deemed as a “benchmark” for further investigation of RILD prognostic models of thoracic tumor.

## Data Availability Statement

The datasets generated for this study will not be made publicly available. The data used in this study was generated in the external validate center. The corresponding author cannot see the data, which was the reason why we performed the distributed learning to avoid data sharing in this study.

## Ethics Statement

The studies involving human participants were reviewed and approved by Velindre Cancer Centre, Cardiff, UK. Written informed consent for participation was not required for this study in accordance with the national legislation and the institutional requirements.

## Author Contributions

ZS implemented the distributed MATLAB code via VLP, converted clinical data of SCOPE1 to RDF format, performed analysis on the results using MATLAB, and made a major contribution to the writing of the manuscript as the first author. KF and TC were responsible for data preparation and quality check of SCOPE1 dataset. JP implemented the imputation analysis to deal with the missing data in SCOPE1 dataset. ES and PW were responsible for VLP setup in Cardiff for distributed learning. JS provided technical support for external validation analysis through VLP. AD and LW acted in the capacity of joint senior authors who motivated the study, set the general methodology and had overall scientific responsibility for this investigation. All co-authors contributed to proof-reading of the manuscript.

### Conflict of Interest

The authors declare that the research was conducted in the absence of any commercial or financial relationships that could be construed as a potential conflict of interest.
