# Supplementary material for: External Validation of Radiation-Induced Dyspnea Models on Esophageal Cancer Radiotherapy Patients
Source: Front Oncol. 2019 Dec 16;9:1411. doi: 10.3389/fonc.2019.01411 (PMC6927468; doi:10.3389/fonc.2019.01411)
Supplement: Supplementary file 1 [file Data_Sheet_1.docx]

**Supplementary - External Validation of Radiation-Induced Dyspnea Models on Esophageal Cancer Radiotherapy Patients**

**Zhenwei Shi ^1^, Kieran Foley ^4^, Juan Pablo de Mey** **^2^, Emiliano Spezi** **^3^, Philip Whybra^3^, Tom Crosby ^4^, Johan van Soest ^1^, Andre Dekker ^1^ and Leonard Wee ^1^**

^1^ Department of Radiation Oncology (MAASTRO Clinic), GROW – School for Oncology and Development Biology, Maastricht University Medical Centre, The Netherlands; ^2^ Faculty of Health Medicine and Life Sciences (FHML), Maastricht University, The Netherlands; ^3^ School of Engineering, Cardiff University, Cardiff, UK; ^4^ Velindre Cancer Centre, Cardiff, UK;

**Table 1: The performance assessment of the validated dyspnea model 1 and model 2 on the external validation cohorts V1 and V2.**

|  | **External validation cohort (V1)** | **External validation cohort (V2)** |
| --- | --- | --- |
| **Incidence** | 11% | 11% |
| **AUC** | 0.68  (95% CI: 0.55-0.76) | 0.70  (95% CI: 0.58-0.77) |
| **Accuracy** | 0.54  (95% CI: 0.47-0.61) | 0.88  (95% CI: 0.84-0.92) |
| **Sensitivity** | 0.70 | 0.32 |
| **Specificity** | 0.52 | 0.95 |
| **PPV** | 0.15 | 0.45 |
| **NPV** | 0.93 | 0.92 |

AUC area under curve; CI confidence interval; PPV positive predictive value; NPV negative predictive value.
